# Traditional Uses of Medicinal Plants in Polonnaruwa District in North Central Province of Sri Lanka

**DOI:** 10.1155/2019/9737302

**Published:** 2019-05-28

**Authors:** Mayuri Tharanga Napagoda, Thamudi Sundarapperuma, Diroshi Fonseka, Sachinthi Amarasiri, Prabath Gunaratna

**Affiliations:** ^1^Department of Biochemistry, Faculty of Medicine, University of Ruhuna, Galle 80000, Sri Lanka; ^2^Faculty of Allied Health Sciences, University of Ruhuna, Galle 80000, Sri Lanka

## Abstract

Sri Lanka harbors over 3000 plant species, and most of these plants have been of immense importance in the traditional systems of medicine in the country. Although there is a rich reserve of indigenous knowledge on medicinal plants, in-depth studies have not been pursued yet to compile the ethnoflora with traditional medicinal applications for the scientific community. Thus, as a continuation of our ethnobotanical inventory work in different regions in the country, the present study was carried out in one of the administrative districts in the North Central area of Sri Lanka known as Polonnaruwa district. The information on the significance of medicinal plants as curative and preventive agents of diseases was collected through semistructured and open-ended interviews from 284 volunteers who were randomly recruited for the study. Ethnobotanical data were analyzed using relative frequency of citation (RFC), family importance value (FIV), and use value (UV). Out of the total participants, 53.7% claimed the use of herbal remedies. A total of 64 medicinal plants belonging to 42 plant families were recorded, out of which *Coriandrum sativum* L. (RFC = 0.163) was the most cited species. Out of the 42 plant families recorded, the FIV was highest in Zingiberaceae. *Coscinium fenestratum* (Goetgh.) Colebr. was found as the plant with the highest use value. Furthermore, the majority of the nonusers of the herbal remedies were willing to adopt herbal products upon the scientific validation of their therapeutic potential. This study revealed that the indigenous herbal remedies are still popular among the local communities in the study area.

## 1. Introduction

Medicinal plants have been used since time immemorial in both developing and developed countries; for example, plants were considered as the material basis of traditional Chinese medicine (TCM) as well as many other ethnic medicine traditions in China [1], while the utilization of medicinal plants as a fundamental component of the African traditional health-care system is believed as the oldest and the most assorted of all therapeutic systems [2]. Similarly, the Indian subcontinent is considered as a vast repository of medicinal plants that have been used in indigenous medical treatments, and even in the present era of modern medicine, traditional health-care systems based on plants and plant-derived products are therapeutically employed on the Indian subcontinent [3]. In the Sri Lankan context, indigenous systems of medicine are widely popular among large segments of the Sri Lankan population despite the influx of modern Western medicine. In general, the traditional systems of medicine available in the country are of four types, namely, Ayurveda, Siddha, Unani, and Deshiya Chikitsa. Plants and plant-based formulations are considered as essential components of the Ayurveda and Deshiya Chikitsa systems [4]. Among the native flora of Sri Lanka, more than 1400 plants are employed for medicinal purposes [5]. Considering the ethnobotanical data in other developing countries in the world, particularly in the neighboring country (India) [6], we could speculate that herbal preparations are more popular among the rural communities in Sri Lanka as well. Hence, a rich reserve of indigenous knowledge of herbal remedies for various ailments is expected to have accumulated especially in the rural areas of the country. The documentation of Sri Lankan medicinal plants to the scientific community was initiated during the colonial period of the country, specially with the descriptions of plant specimens collected by Paul Hermann in the 1670s and also with Icones Plantarum Malabaricarum (1694–1718) [7, 8]. Although these sources represent a rich source of ethnobotanical knowledge from colonial Ceylon, only a handful of ethnobotanical studies have been conducted over the recent years to document the traditional knowledge on medicinal values of plant species used in indigenous medicine [9, 10]. In addition, the book series written by Jayaweera in 1982 on “Medicinal plants (indigenous and exotic) used in Ceylon” [11] are still popular among scientists who are working on medicinal plants and their bioactivities; however, the scientific validation of these traditional claims is still at its infancy. Thus, as a continuation of our ethnobotanical inventory work in different administrative areas in Sri Lanka, the present study was undertaken to assess the significance and contribution of medicinal plants/herbal therapeutics to the day-to-day life of the inhabitants of Polonnaruwa district in the North Central region in Sri Lanka.

As evident from the ethnobotanical studies conducted in other South Asian countries as well as in Africa, the rural communities exploit plants that are easily available in their surroundings for food and medicaments [12–14]. For example, a recent study conducted in Northern Pakistan revealed that the local communities have a rich accessibility of medicinal plants; thus, they opt herbal remedies as low-cost health care for respiratory disorders [15]. Moreover, in the case of herbal therapeutics, people are generally aware about the harmful effects of synthetic medicines, thus realize the importance of a more natural way of life [10]. Moreover, the factors like low financial conditions and unavailability of modern health-care facilities would also limit the access of rural people to synthetic medicines [16]. Hence, the study area for this research has a high potential for utilization and consumption of medicinal plants due to the wide availability of valuable medicinal plants that are unique to the dry zone of Sri Lanka, as well as the presence of rural agricultural communities. Considering all these factors, we hypothesized that the inhabitants in the study area for this research widely utilize medicinal plants as easy and reliable remedies for common disease conditions.

## 2. Materials and Methods

### 2.1. Study Area

**Figure 1:**
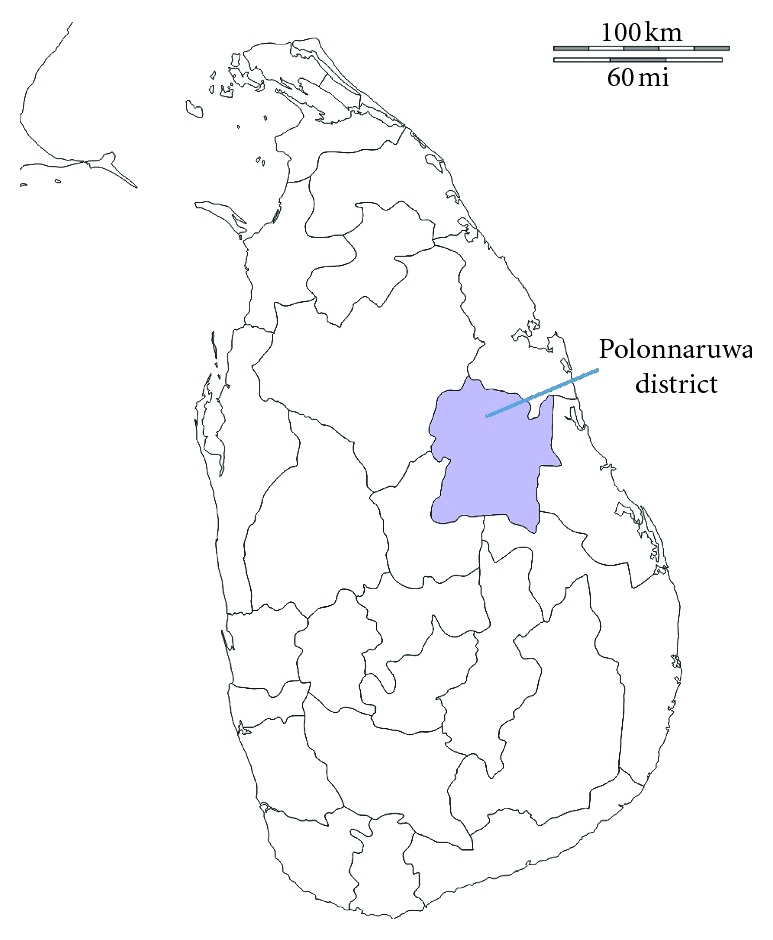
Location of Polonnaruwa district.

Polonnaruwa district is located in the North Central Province of Sri Lanka (Figure 1, Supplementary 1) and has an area of 3,293 km^2^. The district is divided into seven divisional secretariat divisions, which are further subdivided into 295 “Grama Niladhari” divisions. There are 637 villages, and the total population of the district is reported as 403,335. The majority of the people in the district are engaged in agriculture and animal husbandry. The forest coverage, including the grasslands and marshy lands, is estimated as 346,638.2 ha [17]. Twelve government hospitals located within the district provide modern health-care facilities, while 16 Ayurvedic hospitals and a large number of traditional healers within the local communities are responsible for the provision of traditional health-care system.

### 2.2. Ethnobotanical Field Survey and Data Collection

Medicinal plant use was documented in all seven divisional secretariat areas (i.e., Dimbulagala, Elahera, Hingurakgoda, Lankapura, Medirigiriya, Thamankaduwa, Welikanda; see Supplementary 1) in Polonnaruwa district. This survey was carried out from August 2015 to March 2018, and the data were collected from 284 volunteers from the general population of the district who were aged above 30 years, following the method described by Napagoda et al. [10]. In brief, the participants were selected randomly from a list of households in each divisional secretariat area, and visits were made to each of those households for data collection. Informed consent was obtained from each participant in writing prior to the study. A questionnaire was used to collect the information on local name of the plants, source, part(s) used, method of traditional preparation, and some demographic information of the informants such as age, gender, and educational background (Supplementary 2).

The ethical approval was obtained from the ethical review committee, Faculty of Medicine, University of Ruhuna, Sri Lanka. SPSS version 20 was used to recode the collected data.

### 2.3. Plant Specimen Collection and Preservation

Plant species used as herbal remedies were collected, dried, preserved, and mounted on herbarium sheets. The plant materials were identified by one of the authors (MTN), who is a botanist. Botanical names and families were verified using book series titled “Revised Handbook to the Flora of Ceylon” [18] and “Medicinal plants (indigenous and exotic) used in Ceylon” [11]. The botanical names have also been checked with the data available at http://www.theplantlist.org. The specimens were deposited at the Herbarium in the Department of Biochemistry, Faculty of Medicine, University of Ruhuna, Sri Lanka.

### 2.4. Quantitative Analysis of the Ethnobotanical Information

The knowledge on the usage of medicinal plants was quantitatively assessed by the relative frequency of citation (RFC), family importance value (FIV) of a plant family, and use value (UV) as described in our previous study [10] and the method of Kayani et al. [15] by substituting in the relevant equations given below. RFC and FIV were calculated to quantitatively determine the consensus between informants on the use of medicinal plants in the region as it gives the local importance of a species or a family [15, 19, 20].

The value of RFC for a particular species of medicinal plants is based on the citing percentage of informants for that particular species, where RFC = FC/N (0 < RFC < 1), in which RFC is the relative frequency of citation, FC is the number of informants who mentioned the species, and *N* is the total number of informants participating in the study.

Family importance value (FIV) of a plant family was calculated by taking the percentage of informants mentioning the family, where FIV = FC (family)/*N* × 100, in which FC is the number of informants mentioning the plant family and *N* is the total number of informants participating in the study.

Use value indicates the relative importance of plant species known locally, and the following formula was used to determine *UV*: *UV*_*i*_=∑*U*_*i*_/*N*_*i*_, in which *U*_*i*_ is the number of use reports described by each informant for species *i* and *N* is the total number of informants describing the specific species *i*.

## 3. Results and Discussion

As speculated, the results of this study revealed that the majority of the inhabitants who have participated in this study depended on the indigenous plant resources as treatments and preventive measures against a number of disease conditions.

Out of the total of 284 informants, 132 (53.7%) claimed the use of medicinal plants for the treatment of various ailments such as diabetes, inflammatory conditions, and skin diseases, while the rest of the informants (46.3%) mentioned the nonadherence to herbal remedies. In addition, these plants are also used as energy boosters and cosmetics. Among those people, 47.6% firmly believed in the safety and low adverse effects associated with the herbal formulations and mentioned this as a reason for their preference. In addition, the previous success with herbal remedies (35.86%) was also a main contributing factor for the people to continue with plant-based therapies. Unlike the observations of our previous ethnobotanical study conducted in Gampaha District, Western Province of Sri Lanka [10], some people (2.76%) stated that the nonavailability of modern health-care facilities in their villages was a reason for them to opt for herbal remedies. The majority of the users (67.9%) claimed the use of herbal preparations at the initial stage of a disease before going for any other medications, while 26.01% have mentioned the simultaneous usage with other medications. Only 6.1% stated the use of herbal therapeutics as a last resort, when other treatment methods have failed. The knowledge of the herbal remedies had transferred through generations while the influence of media in promoting the use of herbal therapeutics could not be neglected (Table 1).

**Figure 2:**
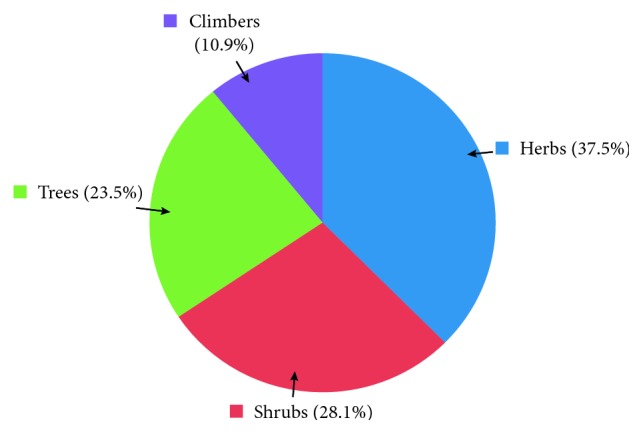
Life form of the plants used as herbal remedies.

**Figure 3:**
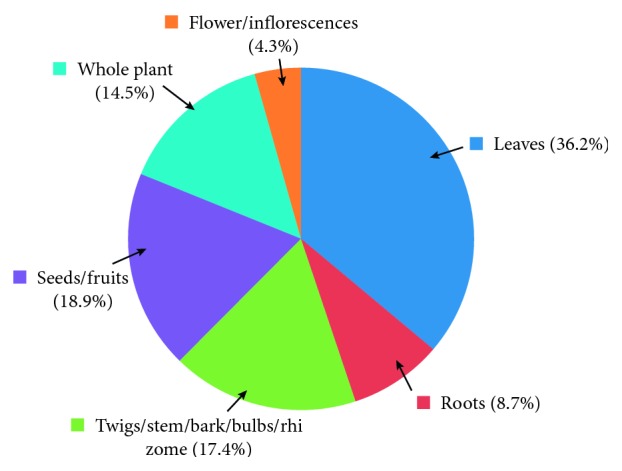
Plant parts used in herbal preparations.

**Figure 4:**
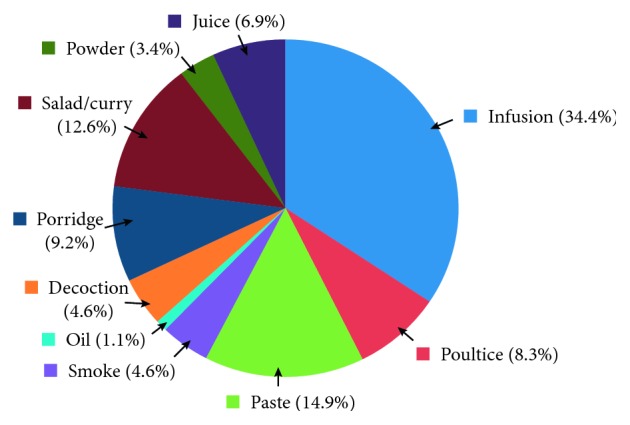
Mode of utilization of plants to treat various disease conditions.

The study revealed the use of 64 medicinal plants belonging to 42 plant families, out of which *Coriandrum sativum* L. (RFC = 0.163) was the most cited species, followed by *Zingiber officinale* Roscoe (RFC = 0.146) and *Hygrophila auriculata* (Schumach.) Heine (RFC = 0.109). The family importance value was highest in Zingiberaceae (22.8%) (Table 2). The highest use value was reported for *Coscinium fenestratum* (Goetgh.) Colebr. The most dominant life form of the species reported was herbs (37.5%; Figure 2). The most frequently used part of the plant was leaves (36.2%; Figure 3), followed by seeds/fruits (18.9%). Medicinal plants used in folk herbal remedies were prepared and administered in various forms. The most common preparation method was infusion (34.4%) while 14.9% were used in the form of a paste (Figure 4). The percentage of oral administration (71.1%) of herbal preparation was much higher than the external or topical application (24.3%) and inhalation (4.6%). Most of the crude drugs were prepared from single plant species; however, combinations of multiple species as well as the use of adjuvants such as honey, sugar, coconut milk, salt, and coconut oil have also been reported. For example, a paste prepared from the fruit of *Myristica fragrans* Houtt. with the juice of *Citrus aurantifolia* (Christm.) Swingle is a common remedy for stomachache while honey or sugar is added to most of the infusions to reduce the bitter taste.

The summary of the medicinal plant species used in Polonnaruwa district to treat various disease conditions is given in Table 3.

**Figure 5:**
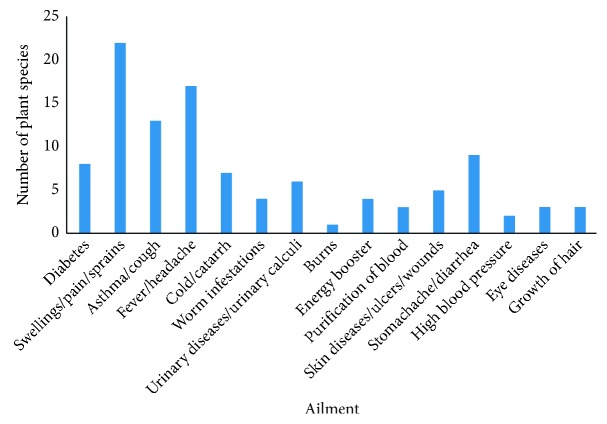
Number of plants used against different disease conditions.

As depicted in Figure 5, herbal remedies were used by the inhabitants of Polonnaruwa district against 15 broad categories of ailments/conditions reporting the highest number of species against swellings/pains or sprains. Further, the local people in the study area utilize medicinal plants (around 30 plant species) for the treatment of other classical inflammatory symptoms like fever [10, 21] or chronic inflammatory diseases like asthma [22].

Interestingly, the medicinal uses of some of the plants mentioned by the informants have not been documented in the literature particularly in the popular book series on Sri Lankan medicinal plants by Jayaweera [11], for example, the use of *Spondias dulcis* Parkinson for high blood pressure and *Hemidesmus indicus* (L.) R. Br. ex Schult., *Artocarpus heterophyllus* Lam, and *Scoparia dulcis* L. for diabetes. Therefore, the documentation of this rich undocumented ethnobotanical knowledge could offer new avenues for pharmacological investigations on prospective new drugs of herbal origin. Moreover, plant species like *Asparagus racemosus* Willd, *Terminalia bellirica* (Gaertn.) Roxb., *Piper betle* L., *Murraya koenigii* (L.) Spreng., *Citrus aurantium* L., *Citrus aurantifolia* (Christm.) Swingle, and *Zingiber officinale* Roscoe have been identified as remedies for snake bites in a recent ethnobotanical study conducted in Western and Sabaragamuwa Provinces in Sri Lanka [9]; however, none of the informants participated in the present study mentioned about the utility of those plants in the treatment of snake bites. Besides, some of the informants mentioned that the wealth of knowledge is rapidly diminishing due to the dearth of elderly people who are knowledgeable on folklore medicine as well as lack of interest in younger generation to systematically study these traditional healing systems. Thus, our findings would enable the preservation of local knowledge which is obtained by trial and error and transferred over generations. In addition, a dramatic degradation of habitat due to construction work and the ruthless use and overexploitation of medicinal plants by local people and the traders of medicinal plants solely for commercial purposes were observed during the field survey. As an example, it has been mentioned that there is a high demand in the local market specially for *Salacia reticulata* Wight, a plant which was also documented in Icones Plantarum Malabaricarum as an endemic species [8]. *S. reticulata* Wight is widely popular among Sri Lankans as an effective remedy for diabetes; thus, there is an increased demand for commercial products prepared from stems of this plant, which could make it highly vulnerable for extinction. Hence, appropriate conservation measures are urgently required to cultivate such valuable medicinal plants and thereby to reduce the pressure on overexploitation from natural habitats. On the other hand, plant species like *Zingiber officinale* Roscoe and *Coriandrum sativum* L. are not threatened by overharvesting despite the high demand, particularly due to the cultivation of *Z. officinale* Roscoe in most of the home gardens throughout the country not only for medicinal but also for culinary purposes as well as the availability of *C. sativum* L. as an imported spice in the local markets in Sri Lanka.

Although the majority of the people in the nonuser category (50.9%) had used some kind of herbal therapeutics at some stage of their lives, the usage was discontinued mainly due to the difficulty in the preparation and collection of plant materials from their surroundings (59%). In addition, the relatively long period of time taken for healing, unpleasant smell, and the taste has also hindered their use. Moreover, some have profusely refused such remedies, due to the unavailability of scientific records on the safety and the efficacy of herbal formulations. Interestingly, 75.4% of these nonusers mentioned that they would shift to herbal products if the efficacy of these products could be scientifically validated.

## 4. Conclusion

This study reports the first in-depth ethnobotanical survey in the North Central Province of Sri Lanka, where agriculture is the primary livelihood of the inhabitants of the area. Among 64 medicinal plants belonging to 42 reported plant families, the family importance value was highest in Zingiberaceae. The most popular medicinal plants among the inhabitants of Polonnaruwa district include *Coriandrum sativum* L., *Zingiber officinale* Roscoe, and *Hygrophila auriculata* (Schumach.) Heine. Despite the eroding folkloric knowledge that depended on the oral tradition for its transmission to successive generations, the indigenous herbal remedies are still popular among the local communities in the study area. Moreover, even the majority of the nonusers are ready to shift to herbal products upon the scientific validation of the therapeutic efficiency, and it signifies the necessity of comprehensive pharmacological and phytochemical investigations of these traditional formulations.

## Figures and Tables

**Table 1:** Statistics on the usage of herbal therapeutics.

Parameter	Percentage
*Demographic data of regular users*	
*Gender*	
Male	59.8
Female	40.2
*Age group (years)*	
30–45	34.09
46–60	38.64
61–75	24.24
>75	3.03
*Educational background*	
University degree/diploma and above	2.3
12 years of school education	15.2
1–11 years of school education	82.5
No schooling	0

*Source of information/knowledge*	
From parents/grandparents	60.42
Neighbours/friends	13.89
Doctors/traditional physicians	7.64
Media	13.19
Own experience	4.86

*Reason for usage*	
Safe/less side effects	47.59
Previous success	35.86
Easy access to the plant materials	13.79
High cost of other treatment methods	0
Nonavailability of modern health-care facilities	2.76

**Table 2:** Family importance value (FIV) of the ten plant families with the highest FIV.

Family	FIV (%)
Zingiberaceae	22.8
Apiaceae	19.9
Acanthaceae	18.3
Rutaceae	12.6
Fabaceae	10.2
Amaranthaceae	9.7
Menispermaceae	9.3
Apocynaceae	8.9
Cucurbitaceae	8.1
Meliaceae	7.7

**Table 3:** Medicinal plant species used in Polonnaruwa district to treat different disease conditions.

Family	Scientific name and voucher specimen number	Vernacular name (in Sinhala)	Life form	Parts used	Preparation	Disease conditions treated	RFC	*UV*	Reported usage in literature [13]
Acanthaceae	*Adhatoda vasica* Nees MN-NCP-01	Adhatoda	Shrub	Leaves, twigs, roots	Infusion, poultice	Swellings in joints, cough, asthma, catarrh	0.073	1.56	Diarrhea, fever, asthma
	*Hygrophila auriculata* (Schumach.) Heine MN-NCP-02	Neermulli	Herb	Whole plant	Infusion, decoction, porridge	Urinary diseases and urinary calculi, headache	0.109	1.1	Oedema, kidney stones, jaundice, rheumatism

Acoraceae	*Acorus calamus* L. MN-NCP-03	Wadakaha	Herb	Root	Infusion, paste made with milk	Cough, worm infestation	0.004	1.0	Asthma, rheumatism, bowel complaints, internal ulceration

Amaranthaceae	*Aerva lanata* (L.) Juss. MN-NCP-04	Polpala	Herb	Whole plant	Infusion, porridge	Urinary diseases, as an energy booster, to purify blood, body pain	0.085	1.57	Kidney stones, cough, headache
	*Alternanthera sessilis* (L.) R. Br. ex DC. MN-NCP-05	Mukunuwenna	Herb	Whole plant	Salad, porridge	Body pain, as an energy booster	0.012	1.67	Liver diseases, acute and chronic pyelitis, snake bites

Amaryllidaceae	*Allium sativum* L. MN-NCP-06	Sudulunu	Herb	Bulb	Infusion, porridge	Asthma stomachache, body pain	0.024	2.5	Asthma, gout

Anacardiaceae	*Spondias dulcis* Parkinson MN-NCP-07	Amberella	Tree	Fruit	Cook with coconut milk	High blood pressure	0.008	1.0	Dysentery, rheumatism, earache

Apiaceae	*Centella asiatica* (L.) Urb. MN-NCP-08	Gotu kola	Herb	Whole plant	Salad, juice, porridge	Catarrh, eye diseases, as an energy booster	0.016	2.0	Kidney diseases, skin diseases, rheumatism, fever, dysentery, pains, epilepsy
	*Coriandrum sativum* L. MN-NCP-09	Koththamalli	Herb	Seeds	Infusion	Cold, fever, asthma, body pain	0.163	1.7	Cold, fever, cough
	*Trachyspermum Roxburghianum* (DC.) H. Wolff MN-NCP-10	Asamodagum	Herb	Leaves	Salad	Stomachache, worm infestation	0.020	1.4	Cough, asthma, dysentery

Apocynaceae	*Hemidesmus indicus* (L.) R. Br. ex Schult. MN-NCP-11	Iramusu	Herb	Root, whole plant	Infusion, porridge	Cold, fever, to purify blood, body pain, diabetes	0.089	1.32	Purification of blood, oedema, skin rashes, cough, asthma

Araceae	*Lasia spinosa* (L.) Thwaites MN-NCP-12	Kohila	Herb	Whole plant	Porridge	As an energy booster	0.004	1.0	Piles

Arecaceae	*Cocos nucifera* L. MN-NCP-13	Kurumba	Tree	Tender coconut water	Drink	Fever	0.004	1.0	Diuretic, anthelmintic

Asparagaceae	*Asparagus racemosus* Willd. MN-NCP-14	Hathawariya	Climber	Whole plant	Infusion	Urinary diseases and urinary calculi	0.024	1.0	Diuretic, dysentery, rheumatism, urinary and kidney diseases

Asphodelaceae	*Aloe vera* (L.) Burm. f. MN-NCP-15	Komarika	Herb	Leaves	Grind to get the juice	Burns, for the growth of hair	0.008	1.5	Swellings, burns, skin diseases, urinary diseases, fever

Asteraceae	*Acanthospermum hispidum* DC. MN-NCP-16	Katu-nerinchi	Herb	Leaves	Paste	Pain in the joints	0.004	1.0	Arthritis, rheumatism, leprosy
	*Eclipta prostrata* (L.) L. MN-NCP-17	Keekirindiya	Herb	Whole plant	Paste	For the growth of hair	0.012	1.0	Skin diseases, ulcers, stimulate the growth of hair, fever, arthritis

Capparaceae	*Cleome gynandra* L. MN-NCP-18	Wela	Herb	Whole plant	Infusion	Pain in joints	0.004	1.0	Arthritis, rheumatism
	*Crateva adansonii* DC. MN-NCP-19	Lunuwarana	Tree	Bark	Decoction	Urinary calculi	0.036	1.0	Urinary calculi

Celastraceae	*Pleurostylia opposita* (Wall.) Alston MN-NCP-20	Panakka	Shrub	Leaves	Salad	Urinary diseases	0.004	1.0	Urinary diseases

Combretaceae	*Terminalia chebula* Retz. MN-NCP-21	Aralu	Tree	Fruit	Powder	Fever	0.016	1.0	Fever, eye diseases, piles, chronic dysentery
	*Terminalia bellirica* (Gaertn.) Roxb. MN-NCP-22	Bulu	Tree	Fruit	Powder	Fever, diarrhea	0.012	1.67	Diarrhea, fever, sore eyes

Costaceae	*Costus speciosus* (J. Koenig) Sm. MN-NCP-62	Thebu	Shrub	Leaves	Salad, infusion	Diabetes	0.020	1.0	Fever, cough, skin diseases

Crassulaceae	*Kalanchoe laciniata* (L.) DC. MN-NCP-23	Akkapana	Herb	Leaves	Infusion	Cough, asthma, cold	0.008	2.0	Urinary diseases, diarrhea, dysentery, cough, cold

Cucurbitaceae	*Coccinia grandis* (L.) Voigt MN-NCP-24	Kowakka	Vine	Leaves	Salad, infusion	Diabetes	0.081	1.0	Diabetes, urinary calculi, skin diseases

Elaeocarpaceae	*Elaeocarpus serratus* L. MN-NCP-25	Veralu	Tree	Tender leaves	Juice	For the growth of hair	0.004	1.0	Dandruff, abscesses, joint swellings

Euphorbiaceae	*Phyllanthus emblica* L. MN-NCP-26	Nelli	Tree	Fruit	Poultice	Redness and swellings in eye	0.016	1.0	Inflammation in eye, gonorrhea, diarrhea, urinary diseases
	*Ricinus communis* L. MN-NCP-27	Enderu	Shrub	Leaves	Poultice	Headache, joint pains, swellings	0.020	1.6	Headache, boils, rheumatism

Fabaceae	Bauhinia racemosa Lam. MN-NCP-28	Maila	Shrub	Leaves	Salad	Urinary diseases	0.004	1.0	Pain, fever, urinary diseases
	*Cassia auriculata* L. MN-NCP-29	Ranawara	Shrub	Flower, leaves	Infusion	Urinary diseases and urinary calculi, to purify blood	0.045	1.27	Fever, diabetes, urinary diseases, rheumatism, eye conjunctivitis, skin diseases
	*Sesbania grandiflora* (L.) Pers. MN-NCP-30	Kathurumurunga	Shrub	Leaves	Salad	Fissuring of lip, ulcers in mouth	0.032	1.0	Oedema, wounds, eye diseases, coughs, fever, skin diseases
	*Tamarindus indica* L. MN-NCP-31	Siyabala	Tree	Leaves	Paste	Swelling in joints	0.020	1.0	Boils, rheumatism

Hippocrateaceae	*Salacia reticulata* Wight MN-NCP-32	Kothala himbutu	Climbing shrub	Stem	Infusion	Diabetes	0.061	1.0	Diabetes, skin diseases, rheumatism

Lamiaceae	*Leucas zeylanica* (L.) W. T. Aiton MN-NCP-33	Gata thumba	Herb	Leaves	Salad	Worm infestation	0.016	1.0	Fever, gout, skin diseases, worm infestation
	*Vitex negundo* L. MN-NCP-60	Nika	Shrub	Leaves	Smoke, paste	Cough, asthma, fever, swellings in joints, cold	0.041	1.4	Rheumatic swellings, headache, catarrh, asthma

Loganiaceae	*Strychnos potatorum* L. f. MN-NCP-34	Ingini	Tree	Seeds	Paste	Swellings in joints	0.008	1.0	Eye diseases, diarrhea

Lythraceae	*Punica granatum* L. MN-NCP-46	Delum	Shrub	Leaves	Infusion to wash eyes	Eye diseases	0.012	1.0	Eye infections, dysentery, cough, asthma, fever

Malvaceae	*Sida acuta* Burm. f. MN-NCP-35	Babila	Herb	Roots	Infusion, decoction	Fever, pain	0.008	1.5	Fever, impotency, rheumatism

Meliaceae	*Azadirachta indica* A. Juss. MN-NCP-36	Kohomba	Tree	Leaves, stem	Poultice, paste, infusion	Pain in joints, itching diabetes, worm infestation	0.077	1.21	Catarrh, leprosy and skin diseases, rheumatism, ulcers and wounds

Menispermaceae	*Coscinium fenestratum* (Goetgh.) Colebr. MN-NCP-37	Veniwelgata	Woody climber	Stem	Infusion	Fever, cough, pain, asthma, skin diseases in children	0.081	2.6	Fever, tetanus, dressing wounds and ulcers
	*Tinospora cordifolia* (Willd.) Miers MN-NCP-38	Rasakida	Climber	Stem	Infusion	Fever	0.012	1.0	Fever, skin diseases, diabetes, dysentery, rheumatism

Moraceae	*Ficus racemosa* L. MN-NCP-39	Attikka	Tree	Fruit	As a curry	Diabetes	0.020	1.0	Urinary diseases, dysentery, diabetes
	*Artocarpus heterophyllus* Lam. MN-NCP-40	Kos	Tree	Root	Infusion	Diabetes	0.004	1.0	Skin diseases, asthma, diabetes, swellings and abscesses

Moringaceae	*Moringa oleifera* Lam. MN-NCP-41	Murunga	Shrub	Bark	Infusion, poultice	Asthma, swellings	0.041	1.3	Asthma, rheumatism, gout, remedy for snake-bite poisoning

Myristicaceae	*Myristica fragrans* Houtt. MN-NCP-42	Sadikka	Shrub	Fruit	Paste prepared with lime juice	Stomachache	0.032	1.0	Nausea, vomiting, stomachache

Piperaceae	*Piper betle* L. MN-NCP-43	Bulath	Climber	Leaves	Paste	Stomachache	0.008	1.0	Cough, antiseptic
	*Piper nigrum* L. MN-NCP-44	Gammiris	Climber	Seeds	Paste	Stomachache	0.012	1.0	Cough, fever, piles

Poaceae	*Eleusine indica* (L.) Gaertn. MN-NCP-45	Belatana	Herb	Whole plant	Poultice	Swellings and sprains	0.024	1.0	Sprains and dislocations

Rubiaceae	*Coffea arabica* L. MN-NCP-47	Kopi	Shrub	Fruit	Infusion	Stomachache	0.049	1.0	Diarrhea, bleeding wounds
	*Ixora coccinea* L. MN-NCP-48	Rathmal	Shrub	Flowers	Infusion	Skin diseases in children	0.004	1.0	Dysentery, reddened eyes and eruptions in children, catarrh

Rutaceae	*Aegle marmelos* (L.) Corrêa MN-NCP-49	Beli	Tree	Leaves, roots, flower	Decoction, infusion	Asthma, fever	0.008	2.5	Fever, asthma, dysentery, piles, dyspepsia,
	*Citrus aurantium* L. MN-NCP-50	Embul dodam	Tree	Fruit	Juice	Cough, to draw out phlegm	0.004	1.0	Chronic cough
	*Citrus aurantifolia* (Christm.) Swingle MN-NCP-51	Dehi	Tree	Leaves	Smoke, juice	Cough, cold, headache, stomachache	0.073	1.22	Cough, stomachache, cleaning wounds, dysentery
	*Murraya koenigii* (L.) Spreng. MN-NCP-52	Karapincha	Shrub	Leaves	Porridge	High blood pressure	0.041	1.0	Constipation, diarrhea, dysentery

Santalaceae	*Santalum album* L. MN-NCP-53	Sudu handun	Shrub	Bark	Paste	Swellings, pain	0.016	1.0	Fever, diarrhea, dysentery, gastric irritation, skin diseases, local inflammation

Sapotaceae	*Madhuca longifolia* (J. Koenig ex L.) J. F. Macbr. MN-NCP-54	Mee	Tree	Seeds	Oil, poultice	Swellings and pain in joints	0.036	1.0	Fractures, rheumatism, snake bites

Scrophulariaceae	Scoparia dulcis L. MN-NCP-55	Wal koththamalli	Herb	Whole plant	Infusion	Diabetes	0.016	1.0	Ear and eye diseases, liver diseases, leprosy, nasopharyngeal infections

Solanaceae	*Solanum xanthocarpum* Schrad. and H. Wendl. MN-NCP-56	Katuwelbatu	Herb	Leaves	Infusion	Fever, cough, asthma	0.053	1.46	Cough, asthma, colic fever, toothache
	*Solanum surattense* Burm. f. MN-NCP-57	Ela batu	Herb	Leaves	Porridge, smoke	Cough, asthma	0.004	2.0	Rheumatism, cough, diarrhea

Theaceae	*Camellia sinensis* (L.) Kuntze MN-NCP-58	Tea	Shrub	Leaves	Infusion	Stomachache	0.008	1.0	Catarrh, urinary diseases

Verbenaceae	*Lantana camara* L. MN-NCP-59	Gandapana	Shrub	Leaves	Smoke	Fever, cough, asthma	0.008	1.5	Asthma, fever, cough

Zingiberaceae	*Alpinia galanga* (L.) Willd. MN-NCP-61	Araththa	Herb	Rhizome	Infusion	Fever	0.057	1.0	Rheumatism, bronchitis
	*Curcuma longa* L. MN-NCP-63	Kaha	Herb	Rhizome	Paste, powder	Wounds, skin diseases, sprains	0.024	1.5	Sprains, wounds, dysentery, jaundice, rheumatism, skin diseases
	*Zingiber officinale* Roscoe MN-NCP-64	Inguru	Herb	Rhizome	Infusion	Fever, cold, asthma, cough	0.146	1.44	Cold, cough, fever, asthma

## Data Availability

The data used to support the findings of this study are included within the article.

## Abbreviations

RFC: Relative frequency of citation
FIV: Family importance value
UV: Use value.

## Supplementary Materials

Supplementary Materials Supplementary 1: the map of Polonnaruwa district (the district boundary is marked in purple, and the sites where the data were collected are marked with squares). Supplementary 2: the questionnaire which was used to collect the information on utility of herbal preparations and some demographic information of the informants.

## References

[1] Zhang L., Zhuang H., Zhang Y. (2018). Plants for health: an ethnobotanical 25-year repeat survey of traditional medicine sold in a major marketplace in North-West Yunnan, China. *Journal of Ethnopharmacology*.

[2] Mahomoodally M. F. (2013). Traditional medicines in Africa: an appraisal of ten potent African medicinal plants. *Evidence-Based Complementary and Alternative Medicine*.

[3] Pandey M. M., Rastogi S., Rawat A. K. S. (2013). Indian traditional ayurvedic system of medicine and nutritional supplementation. *Evidence-Based Complementary and Alternative Medicine*.

[4] Weragoda P. B. (1980). The traditional system of medicine in Sri Lanka. *Journal of Ethnopharmacology*.

[5] Wijesundera D. S. A. (2004). Inventory, documentation and medicinal plant research in Sri Lanka. *Medicinal Plant Research in Asia*.

[6] Unial A. K., Singh C., Singh B., Kumar M., da Silva J. A. T. (2011). Ethnomedicinal use of wild plants in Bundelkhand Region, Uttar Pradesh, India. *Journal of Medicinal and Aromatic Plant Science and Biotechnology*.

[7] van Andel T., Barth N. (2011). Paul Hermann’s ceylon herbarium (1672–1679) at Leiden, the Netherlands. *Taxon*.

[8] van Andel T., Scholman A., Beumer M. (2018). Icones plantarum malabaricarum: early 18th century botanical drawings of medicinal plants from colonial Ceylon. *Journal of Ethnopharmacology*.

[9] Dharmadasa R. M., Akalanka G. C., Muthukumarana P. R. M., Wijesekara R. G. S. (2016). Ethnopharmacological survey on medicinal plants used in snakebite treatments in Western and Sabaragamuwa provinces in Sri Lanka. *Journal of Ethnopharmacology*.

[10] Napagoda M. T., Sundarapperuma T., Fonseka D., Amarasiri S., Gunaratna P. (2018). An ethnobotanical study of the medicinal plants used as anti-inflammatory remedies in Gampaha District-Western Province, Sri Lanka. *Scientifica*.

[11] Jayaweera D. M. A. (1982). *Medicinal Plants (Indigenous and Exotic) used in Ceylon, Part 1–5*.

[12] Namsa N. D., Tag H., Mandal M., Kalita P., Das A. K. (2009). An ethnobotanical study of traditional anti-inflammatory plants used by the Lohit community of Arunachal Pradesh, India. *Journal of Ethnopharmacology*.

[13] Umair M., Altaf M., Abbasi A. M. (2017). An ethnobotanical survey of indigenous medicinal plants in Hafizabad district, Punjab-Pakistan. *PLoS One*.

[14] Towns A. M., Ruysschaert S., van Vliet E., van Andel T. (2014). Evidence in support of the role of disturbance vegetation for women’s health and childcare in Western Africa. *Journal of Ethnobiology and Ethnomedicine*.

[15] Kayani S., Ahmad M., Zafar M. (2014). Ethnobotanical uses of medicinal plants for respiratory disorders among the inhabitants of Gallies—Abbottabad, Northern Pakistan. *Journal of Ethnopharmacology*.

[16] Diallo D., Hveem B., Mahmoud M. A., Berge G., Paulsen B. S., Maiga A. (1999). An ethnobotanical survey of herbal drugs of Gourma district, Mali. *Pharmaceutical Biology*.

[17] Department of Census and Statistics (2016). *District Statistical Hand Book Polonnaruwa*.

[18] Dassanayake M. D., Fosberg F. R. (1980-2000). *A Revised Handbook to the flora of Ceylon*.

[19] Šavikin K., Zdunić G., Menković N. (2013). Ethnobotanical study on traditional use of medicinal plants in South-Western Serbia, Zlatibor district. *Journal of Ethnopharmacology*.

[20] Vitalini S., Iriti M., Puricelli C., Ciuchi D., Segale A., Fico G. (2013). Traditional knowledge on medicinal and food plants used in Val San Giacomo (Sondrio, Italy)–an alpine ethnobotanical study. *Journal of Ethnopharmacology*.

[21] Ferrero-Miliani L., Nielsen O. H., Andersen P. S., Girardin S. E. (2007). Chronic inflammation: importance of NOD2 and NALP3 in interleukin-1beta generation. *Clinical and Experimental Immunology*.

[22] Ishmael F. T. (2011). The inflammatory response in the pathogenesis of asthma. *Journal of the American Osteopathic Association*.

