# Supplementary material for: Traditional Uses of Medicinal Plants in Polonnaruwa District in North Central Province of Sri Lanka
Source: Scientifica (Cairo). 2019 May 28;2019:9737302. doi: 10.1155/2019/9737302 (PMC6558606; doi:10.1155/2019/9737302)
Supplement: Supplementary Materials — Supplementary 1: the map of Polonnaruwa district (the district boundary is marked in purple, and the sites where the data were collected are marked with squares). Supplementary 2: the questionnaire which was used to collect the information on utility of herbal preparations and some demographic information of the informants. [file 9737302.f1.pdf]

Supplementary-1

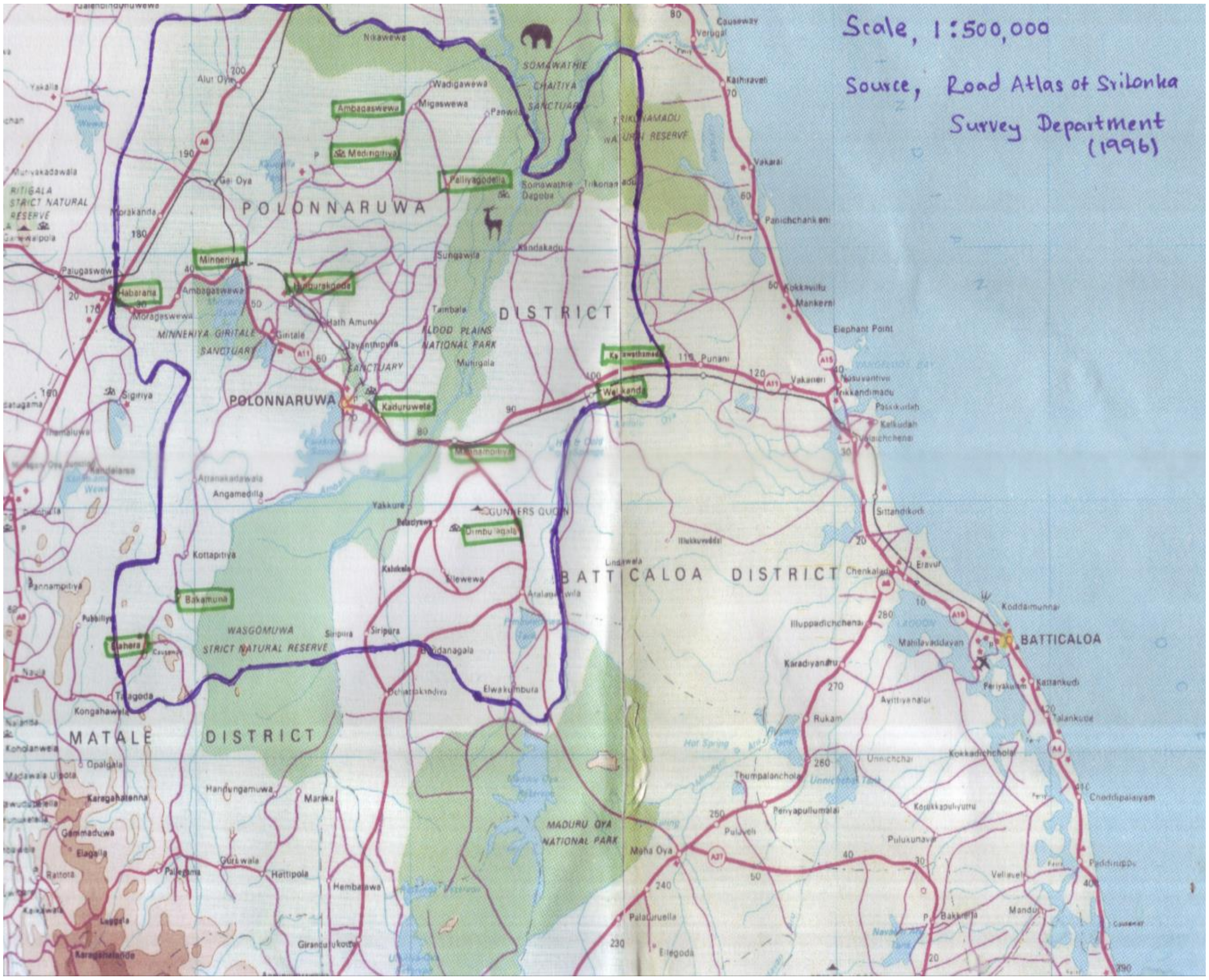

<u>Supplementary-2</u>

Questionnaire

<u>Personal Information</u>

Location : Divisional Secretary's Division:

Age (Years) : 30-45 ☐ 46-60 ☐ 61-75 ☐ above 75 ☐

Gender : Male ☐ Female ☐

Education : Not attended school ☐ 1-11 years of school education ☐

12 years of school education ☐ Diploma/ Degree and above ☐

<u>Medicinal plant usage</u>

Que.1. Are you normally using any medicinal plant/plant preparation for any disease condition?

Yes ☐ **Please answer Que.2**

No ☐ **Please answer Que.3**

Que.2. (If you answered "Yes" to Que.1)

(2.1) **Why do you use herbal therapeutics ?**

I feel that these are safe/ have less side effects. ☐

I had previous success with these. ☐

Plant materials are readily available. ☐

I could not bear the cost of other treatment methods. ☐

Modern health care facilities are not available in my area. ☐

I feel that other treatment methods could not cure my disease ☐

Other reasons (please specify)…………………………………………………………………………………

(2.2) **At what stage/s of the disease you go for these treatments?**

Initial stage before going for any other medication ☐

Simultaneously with other medications ☐

In convalescence period ☐

At the last stage, when other treatment methods fail ☐

(2.3) **Since how many years you are using herbal therapeutics ?** ……………………………….

(2.4) Please provide information to fill the following table:

Disease condition	Name of the plant	Plant part/s used	Preparation method	Administration route	Are you using any other additives ? Eg. Honey, sugar,salt, ghee etc.	How do you obtain the plant material ? Eg. Grown in the garden, purchase, collect from wild etc

(2.5) **How did you acquire the knowledge on herbal therapeutics?**

From ancestors ☐ From neighbors/friends ☐

From traditional physicians ☐ From my own experiences ☐

From audio/visual sources ☐ Others (please specify)………………………

Que.3. (If you answered "No" to Que.1)

(3.1) **Have you ever used any kind of herbal therapeutics for the above mentioned disease conditions at any time of your life ?** Yes ☐ No ☐

(3.2) **Why you are not using herbal therapeutics ?**

It takes more time to get cured ☐

It didn't work well when I used them earlier ☐

It's difficult to find the plant materials ☐

It's difficult to prepare ☐

I don't like the taste/smell ☐

I don't have any faith on these because scientifically not proven ☐

Other reasons (please specify) ………………………………………………………………

(3.3) **Do you think that you would go for herbal therapeutics, if proper scientific investigations are done on them to prove the safety and efficacy?**

Yes ☐ No ☐ Not sure ☐
